# Microangiopathic Hemolytic Anemia Is a Late and Fatal Complication of Gastric Signet Ring Cell Carcinoma: A Systematic Review and Case-Control Study

**DOI:** 10.1093/oncolo/oyac093

**Published:** 2022-05-19

**Authors:** Robert Lam, Nicholas Tarangelo, Rong Wang, Masayasu Horibe, Alyssa A Grimshaw, Dhanpat Jain, Samir Haffar, Fateh Bazerbachi, Pamela L Kunz, Darrick K Li

**Affiliations:** Section of Digestive Diseases, Yale School of Medicine, New Haven, CT, USA; Section of Digestive Diseases, Yale School of Medicine, New Haven, CT, USA; Department of Chronic Disease Epidemiology, Yale School of Public Health, New Haven, CT, USA; Division of Gastroenterology and Hepatology, Keio University School of Medicine, Shinjuku-ku, Tokyo, Japan; Harvey Cushing/John Hay Whitney Medical Library, Yale School of Medicine, New Haven, CT, USA; Department of Pathology, Yale School of Medicine, New Haven, CT, USA; Gastroenterology Department, Syrian Specialty Hospital, Damascus, Syrian Arab Republic; CentraCare, Interventional Endoscopy Program, Saint Cloud Hospital, St. Cloud, MN, USA; Section of Medical Oncology, Department of Medicine, Yale Cancer Center, Yale School of Medicine, New Haven, CT, USA; Section of Digestive Diseases, Yale School of Medicine, New Haven, CT, USA

**Keywords:** signet ring, microangiopathic hemolytic anemia, gastric cancer, systematic review, case-control study

## Abstract

**Background:**

Microangiopathic hemolytic anemia (MAHA) is a rare paraneoplastic syndrome that has been reported in patients with gastric signet ring cell carcinoma (SRCC). Clinical and prognostic features of MAHA in this setting have been poorly described.

**Materials and Methods:**

We conducted a systematic review in 8 databases of gastric SRCC complicated by MAHA and performed a case-control study assessing factors associated with survival in patients with gastric SRCC and MAHA in our pooled cohort compared with age-, sex-, and stage-matched cases of gastric SRCC from the Surveillance, Epidemiology, and End Results (SEER) database. Descriptive analyses were performed and multivariable Cox-proportional hazards regression modeling was used to determine factors associated with overall survival.

**Results:**

All identified patients (*n* = 47) were symptomatic at index presentation, commonly with back/bone pain, and dyspnea. Microangiopathic hemolytic anemia was the first manifestation of gastric SRCC in 94% of patients. Laboratory studies were notable for anemia (median 7.7 g/dL), thrombocytopenia (median 45.5 × 10^3^/μL), and hyperbilirubinemia (median 2.3 mg/dL). All patients with MAHA had metastatic disease at presentation, most often to the bone, bone marrow, and lymph nodes. Median survival in patients with gastric SRCC and MAHA was significantly shorter than a matched SEER-derived cohort with metastatic gastric SRCC (7 weeks vs 28 weeks, *P* < .01). In multivariate analysis, patients with MAHA were at significantly increased risk of mortality (HR 3.28, 95% CI 2.11-5.12).

**Conclusion:**

Microangiopathic hemolytic anemia is a rare, late-stage complication of metastatic gastric SRCC and is associated with significantly decreased survival compared with metastatic gastric SRCC alone.

## Teaser

Microangiopathic hemolytic anemia (MAHA) is a rare paraneoplastic syndrome that has been reported in patients with gastric signet ring cell carcinoma. This review assesses the clinical and prognostic features of MAHA in this setting.

**Implications for Practice**

Microangiopathic hemolytic anemia (MAHA) is a rare complication in cases of gastric signet ring cell carcinoma (SRCC). This article presents findings from a systematic review of all published cases of MAHA associated with gastric SRCC and a case-control study from a matched cohort of gastric SRCC. The findings highlight the importance of recognizing the clinical phenotype of MAHA and the need to rule out underlying occult gastric SRCC. In addition to high-yield workup and management recommendations for clinicians, our study provides valuable prognostic data that can be shared with patients and their families.

## Introduction

Gastric cancer is the third leading cause of cancer-related death worldwide.^1^ Signet-ring cell carcinoma (SRCC) is a rare subtype that accounts for ~15% of gastric adenocarcinoma cases and is characterized histologically by the presence of distinct tumor cells with mucinous cytoplasm and crescent-shaped nucleus.^2-4^ Signet-ring cell carcinoma tends to have an aggressive course and commonly presents at an advanced stage.^5,6^

Microangiopathic hemolytic anemia (MAHA) is a rare paraneoplastic syndrome described in patients with solid tumors, including metastatic SRCC. Tumor cell emboli, immune complexes, and tumor-derived factors are thought to contribute to the Coombs-negative hemolytic process that occurs.^7^ The diagnosis is made when schistocytes are identified on peripheral blood smear.^8^ The differential diagnosis for MAHA includes disseminated intravascular coagulation, thrombotic thrombocytopenic purpura, hemolytic uremic syndrome, heparin-induced thrombocytopenia, malignant hypertension, HELLP (hemolysis, elevated liver enzymes, and low platelets) syndrome, antiphospholipid syndrome, and drug-induced thrombotic microangiopathy.^9^

Familiarity with the clinical features of this rare complication including laboratory testing, radiologic findings, and treatment response may lead to prompt and accurate diagnosis and prognostication. To this end, we report a case of metastatic gastric SRCC complicated by MAHA according to CARE guidelines,^10^ perform a systematic review and synthesis of individual patient data of reported cases, and compare gastric SRCC complicated by MAHA to age-, sex-, and stage-matched gastric SRCC in general via regression analysis incorporating data from the Surveillance, Epidemiology, and End Results (SEER) database.

## Case Presentation

A 36-year-old previously healthy woman was admitted to a major academic medical center after a presyncopal episode associated with nausea, vomiting, and diaphoresis. She also endorsed a 1-week history of new lower back pain. She denied any signs of gastrointestinal bleeding. Initial vital signs were temperature 100.5°F, blood pressure 107/45, respiratory rate 16/minute, and SaO_2_ of 100% on room air. Physical examination was notable for tenderness in the lumbar area with no neurologic abnormalities.

Laboratory results were notable for hemoglobin 6.8 g/dL, MCV 97.6 fL, platelets 116 × 10^3^/μL, total bilirubin 3.4 mg/dL, direct bilirubin 0.3 mg/dL, LDH 729 U/L, alkaline phosphatase 204 U/L, AST 64 U/L, ALT 34 U/L, and reticulocyte count >18%, consistent with a consumptive hemolytic process. Coagulation studies included normal prothrombin time 11.5 s, partial thromboplastin time 25.4 s, and fibrinogen 424 mg/dL. Peripheral blood smear showed schistocytes and macrocytic anemia. A direct Coombs test was negative. Additional workup was negative for antiphospholipid syndrome, lupus anticoagulant, and glucose-6-phosphate dehydrogenase deficiency.

**Figure 1.**
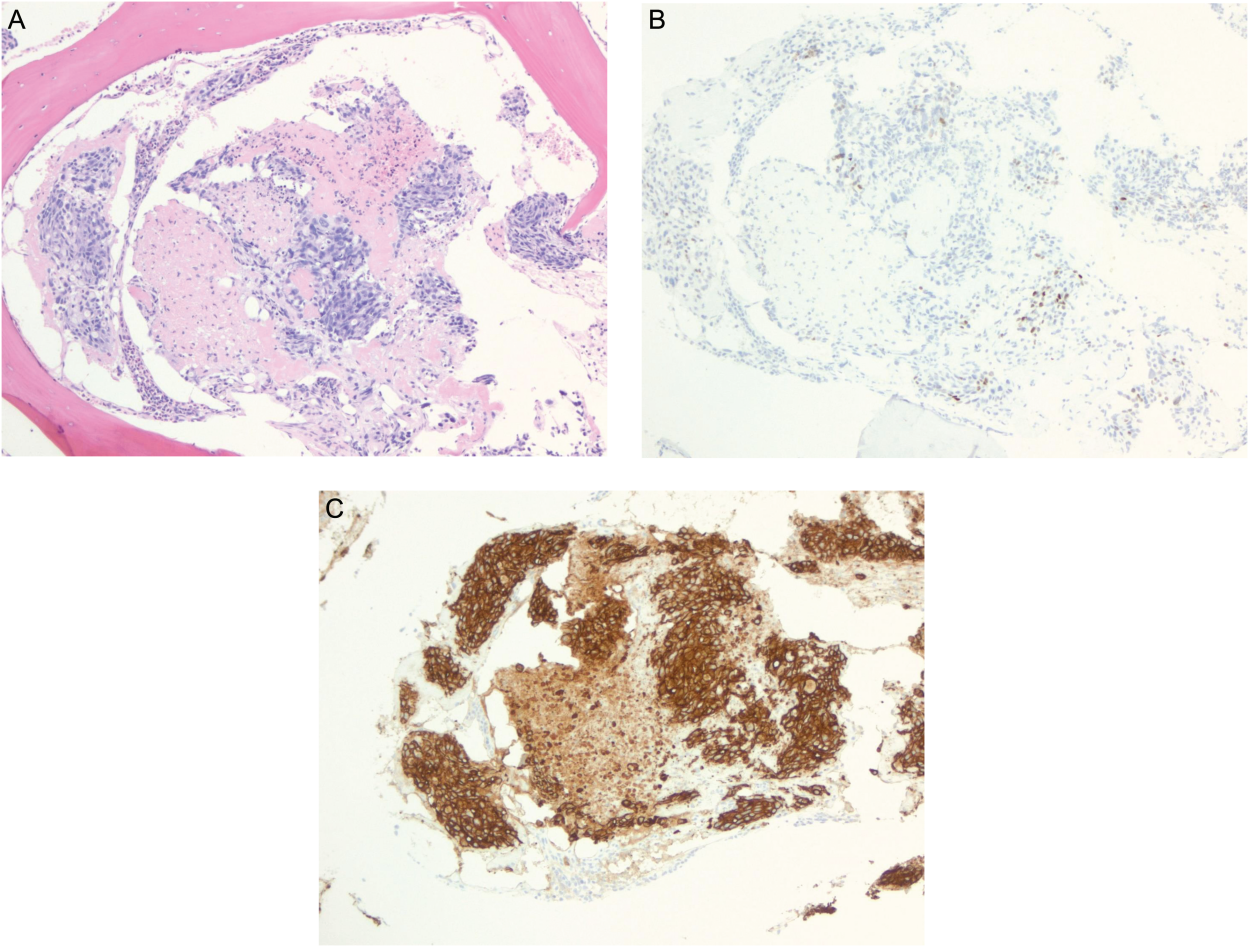
Bone biopsy of metastatic L1 lytic lesion. (**A**) Areas of poorly differentiated adenocarcinoma with necrosis (H&E stain, ×100). (**B**) Scattered CX2-positive nuclei (CX2 stain, ×100). (**C**) Diffuse keratin AE1/AE3 positivity (keratin AE1/AE3 stain, ×100); the tumor cells were also positive for CK7 and CK20.

Although lumbar radiographs were normal, computerized tomography (CT) scan of the chest, abdomen, and pelvis identified supraclavicular lymphadenopathy and lytic lesions of the L1 vertebral body with endplate compression fractures. Magnetic resonance imaging (MRI) of the spine, abdomen, and pelvis revealed multifocal enhancing osseous lesions throughout the spine consistent with metastatic cancer. Tumor markers CEA and CA19-9 were elevated to 16.3 ng/mL and 4819 U/mL, respectively. Image-guided bone biopsy of the L1 lytic lesion was notable for bone marrow infiltrated by poorly differentiated carcinoma cells that had intracytoplasmic mucin vacuoles and ill-formed glandular structures (Fig. 1A). The tumors cells were positive for AE1/AE3, CK7, CK20, and CDX2 immunostains, but negative for GATA3, PAX8, CD20, and CD3; this was consistent with metastatic carcinoma of gastrointestinal origin (Fig. 1B, C). A bone marrow biopsy showed marked hypocellularity and highly necrotic tumor positive for AE1/AE3, similar to the bone biopsy lesion with metastatic carcinoma (Supplementary Fig. S1A-C). The tumor was negative for Her2 and PDL-1.

**Figure 2.**
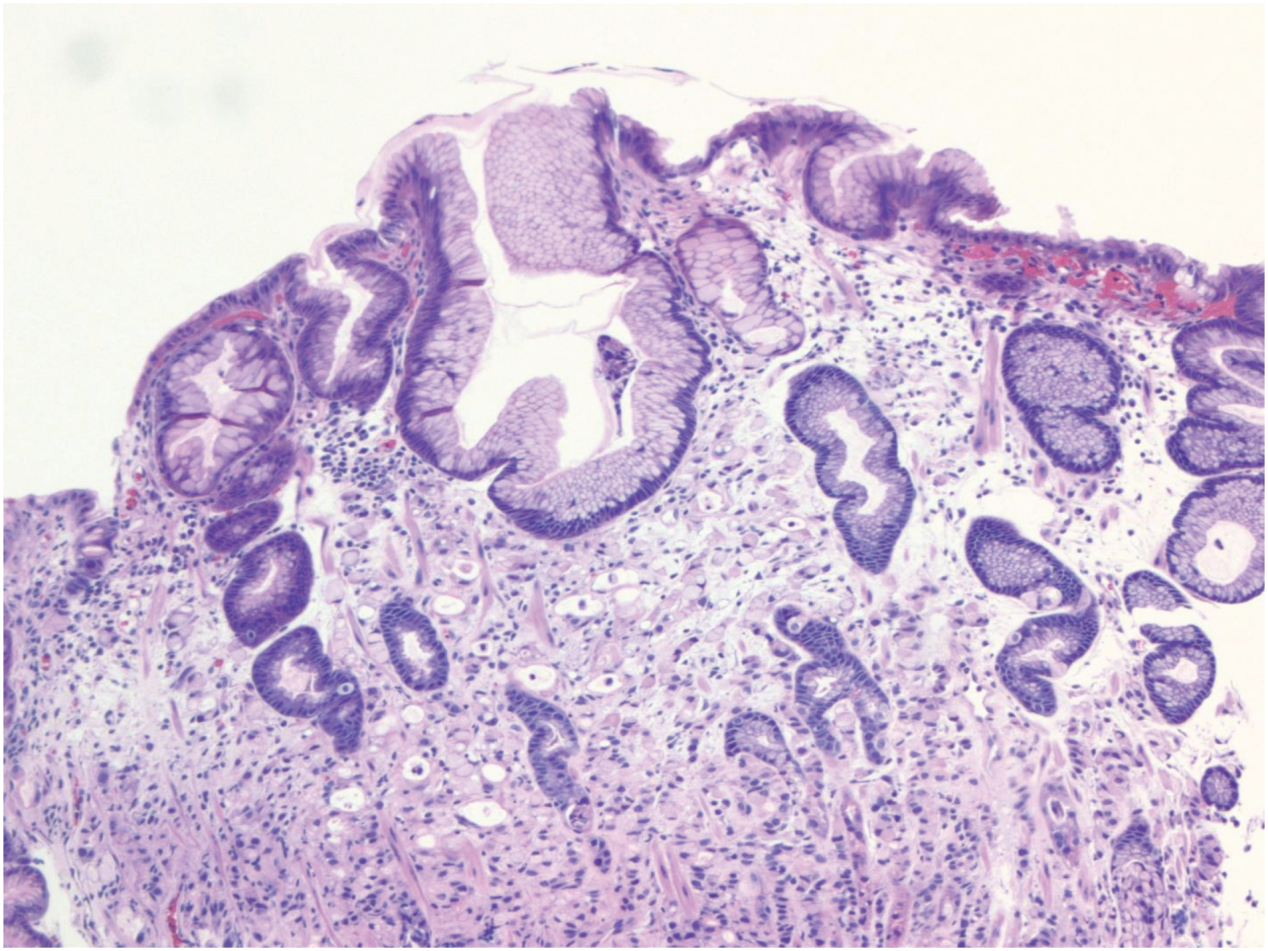
Gastric biopsy of non-bleeding ulcer. Poorly differentiated adenocarcinoma infiltrating into the lamina propria with signet ring cells characterized by cells showing a cytoplasmic mucin vacuole and nucleus pushed to the periphery (H&E stain, ×200).

Given the bone biopsy findings, upper endoscopy and colonoscopy was performed which showed a non-bleeding ulcer in the gastric body (Supplementary Fig. S2) with a normal colon. Biopsies from the gastric ulcer showed a poorly differentiated adenocarcinoma with signet ring cell features (Fig. 2). Primary breast cancer was ruled out with a normal BIRADS-1 (Breast Imaging Reporting and Data System) breast ultrasound and mammogram. The patient was diagnosed with MAHA secondary to gastric SRCC. Treatment of spinal metastases was initiated with dexamethasone and palliative radiation to the cervical and lumbar spine. During her index hospitalization, she received one cycle of dose-reduced FOLFOX (fluorouracil [5-FU], leucovorin, oxaliplatin) with prolonged hospitalization complicated by bilateral spontaneous subdural hematomas with midline shift and altered mental status. She was ultimately discharged after 30 days of hospitalization and completed an second cycle of FOLFOX as an outpatient.

Unfortunately, despite cancer-directed treatment and aggressive supportive care, the patient became transfusion-dependent, and her bony pain became increasingly severe, requiring rehospitalization. Interval radiographs of the patient’s pelvis and femur confirmed extensive metastatic disease, and MRI of the lumbar spine demonstrated new pathologic L4 vertebral body compression fractures. Given the patient’s widespread metastatic disease, evidence of progression, poor quality of life, and grim prognosis, she was transitioned to comfort measures and died 10 weeks after her initial diagnosis.

## Methods

### Systematic Review

The systematic review is reported according to the guidance of the preferred reporting items for systematic reviews and meta-analysis (PRISMA) statement and statement for reporting and synthesis without meta-analysis with an a priori protocol (Supplementary Tables S1-S3).^11,12^ The study was registered in PROSPERO as study CRD42021240057. Considering the rarity of this paraneoplastic phenomenon, we used a mixed methods design that combines health systems data with a systematic review of the literature to identify the largest number of unique cases as recommended by the methods guide of the Agency for Healthcare Research and Quality.^13^

#### Search Strategy and Data Sources

A systematic search was conducted by a medical librarian (A.G.) in Cochrane Library, EMBASE, Google Scholar, Japan Medical Abstracts Society, Ovid MEDLINE, PubMed, Scopus, and Web of Science Core Collection databases using various structured terms and text words without language restriction for studies reporting cases of signet cell carcinoma and MAHA on March 2, 2021. Reference lists of relevant papers were also screened manually for additional cases. Details regarding the search strategy are provided in Supplementary Table S4. Inclusion criteria included adult patients aged 18 or older with biopsy-confirmed gastric SRCC and laboratory evidence of MAHA. We excluded duplicate cases or cases in which there was insufficient reporting of clinical data of individual patients (Supplementary Table S5). Japanese studies were extracted by a native Japanese clinician (M.H.). Other non-English studies were translated with Google Translate (https://translate.google.com) with assistance from a native language speaker, if needed.

The methodological quality and synthesis of case series and case reports tool was used to evaluate included reports (Supplementary Table S6), and this tool has been applied previously with consistency among reviewers.^14-16^ Study selection, data extraction, and risk of bias assessment were made by 2 independent reviewers (R.L., N.T.), and disagreements were settled by discussion and adjudication by the corresponding author (D.K.L.). Study selection was conducted using Covidence (https://www.covidence.org/), and data extraction was completed in REDCap (https://www.project-redcap.org/). The data extracted included year of publication, publication format, country of origin, publication language, age, sex, ethnicity, medical history, clinical symptoms at presentation, laboratory findings, endoscopic findings, radiological exams, treatment, post-treatment complications, duration of follow-up after therapy, final outcome, and cause of death.

### Surveillance, Epidemiology, and End Results Database

#### Patient Selection

The SEER database is an open public database launched by the National Cancer Institute in 1973. It is the authoritative source of clinical information regarding cancer patients in the US. We used the SEER 18 database, which includes a full set of 18 registries and has the broadest coverage of clinical data between years 2000 and 2018.

Eligible controls selected from the SEER database had to be adults aged ≥19 with a diagnosis of distant signet ring gastric cancer (ICD-O3: histology 8490/3 and site 16.x) between 2000-2017. We excluded patients who: (1) were diagnosed with cancer-based on autopsy or death certificate, (2) were alive with no survival time data, and (3) another cancer diagnosis. A total of 6607 newly diagnosed distant SRCC of gastric origin were identified as potential controls in the final SEER cohort. SEER*Stat 8.3.9.2 was used to identify patients.

#### Variables

Among eligible patients, we extracted data for the following covariates: sex, race, age at diagnosis, year of diagnosis, survival time, survival status, and treatment data (no treatment, chemotherapy only, other treatment). The outcome of interest was overall survival, as defined from diagnosis to death or last follow-up visit.

#### Matching Process

Our pooled cohort of patients was matched to controls from the SEER database in a 1:4 ratio based on sex, age, and year of diagnosis. Up to 4 years of variation between cases and matched controls were permitted to minimize era-specific differences in management.

#### Statistical Analysis

For descriptive analysis, we reported medians and ranges for continuous variables and percentages for dichotomized variables unless otherwise specified. A comparison of outcomes was performed using Pearson’s *χ*^2^ test. When the number of patients in any cell in a contingency table was under 5, we used Fisher’s exact test. Two-tailed *P*-values were statistically significant when they were below 0.05. Multivariable Cox regression models were used to estimate hazard ratios (HR) and 95% confidence intervals (CI) for risk factors related to overall survival. Kaplan-Meier analysis was used to estimate cumulative survival probability. All analyses were performed using SAS (Version 9.4, SAS Institute, Cary, NC).

## Results

### Study Characteristics

**Figure 3.**
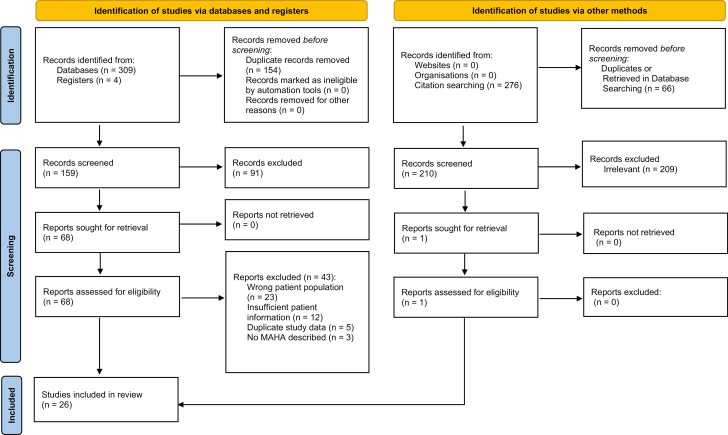
Flow diagram of study selection. Adapted from Page et al.^11^. For more information, visit: http://www.prisma-statement.org/.

A flow diagram of the study selection in shown in Fig. 3. A total of 26 publications between 1985 to 2021 were identified that met the study selection criteria. Eighteen publications were in English, 5 in Japanese, 2 in German, and one in Spanish. Details regarding the countries of origin for reach publication can be seen in Supplementary Table S7.

### Assessment of Methodological Quality of Included Studies

Reviewer agreement in the methodological quality of included studies was 100%. Most studies showed an unclear risk for selection bias. There was low risk of ascertainment, causality, and reporting bias (Supplementary Table S8).

### Demographics and Baseline Laboratory Studies

We identified 47 patients with SRCC of gastric origin and MAHA (Table 1). Median age was 48 (range 19-83), and patients were of White (58.7%) or Asian (41.3%) race. All patients had symptoms at the time of evaluation, most commonly back pain (34%), bone pain (29.8%), and dyspnea (21.3%). MAHA was present at the initial diagnosis of the gastric SRCC in 44 patients (93.6%). Laboratory studies were consistent with a consumptive, hemolytic process, specifically low hemoglobin (median 7.7 g/dL, IQR 2.2 g/dL), low platelets (median 45.5 × 10^3^/μL, IQR 28.0x10^3^/μL), elevated total bilirubin (median 2.3 mg/dL, IQR 2.6 mg/dL), low haptoglobin (median 0 g/L, IQR 0.01 g/L) and high lactate dehydrogenase (median 774 IU/L, IQR 1184 IU/L). In 7 cases, carcinoembryonic antigen (CEA) was checked and was found to be markedly elevated to median 23.4 ng/mL (IQR 63.25 ng/mL). In 29 patients for which the type of MAHA was further subclassified, MAHA was classified as disseminated intravascular coagulation (DIC) and thrombotic thrombocytopenic purpura (TTP) in 86.2% and 13.8% of patients, respectively. A detailed description of baseline laboratory values, treatment and outcome data for each patient is provided in Supplementary Table S9.

**Table 1.** Summary of baseline characteristics of pooled patient cohort with gastric signet ring cell carcinoma and microangiopathic hemolytic anemia.

Pooled cohort		
	Number of patients	Results
Age, median (IQR)	47	48 (25.5)
Male sex, *n* (%)	47	29 (61.7%)
Race, *n* (%)		
White	46	27 (58.7%)
Asian		19 (41.3%)
Ethnicity		
Hispanic	47	4 (8.5%)
Non-Hispanic		43 (91.5%)
Clinical symptoms		
Presence of symptoms at initial evaluation, *n* (%)	47	47 (100%)
Presenting symptom, *n*(%)	47	
Back pain		16 (34%)
Bone pain		14 (29.8%)
Dyspnea		10 (21.3%)
Symptomatic anemia		17 (36.2%)
Weight loss		7 (14.9%)
Abdominal pain		5 (10.6%)
Nausea		3 (6.3%)
Vomiting		4 (8.5%)
Gastrointestinal bleeding		2 (4.2%)
MAHA as first presentation of gastric SRCC	47	44 (93.6%)
Laboratory data		
Hemoglobin, median (IQR)	35	7.7 g/dL (2.2 g/dL)
Platelets, median (IQR)	39	45.5 × 10^3^/μL (28.0 × 10^3^/μL)
White blood cell count, median (IQR)	29	11.2 × 10^3^/μL (6.4 × 10^3^/ μL)
Aspartate transaminase (AST), median (IQR)	9	68 units/L (64 units/L)
Alanine transaminase (ALT), median (IQR)	8	54.5 units/L (51 units/L)
Alkaline phosphatase, median (IQR)	12	589 IU/L (546.5 IU/L)
Total bilirubin, median (IQR)	25	2.3 mg/dL (2.6 mg/dL)
Direct bilirubin, median (IQR)	9	0.7 mg/dL (0.8 mg/dL)
Haptoglobin, median (IQR)	8	0 g/L (0.01 g/L)
Lactate dehydrogenase (LDH), median (IQR)	29	774 IU/L (1184 IU/L)
Creatinine, median (IQR)	11	0.8 mg/dL (0.125 mg/dL)
BUN, median (IQR)	8	25 mg/dL (15.5 mg/dL)
International normalized ratio (INR), median (IQR)	7	1.42 (0.3)
Carcinoembryonic antigen (CEA), median (IQR)	7	23.4 ng/mL (63.25 ng/mL)

Abbreviation: IQR, interquartile range.

### Diagnostic Workup and Findings

The most used diagnostic studies included peripheral blood smear (70.2%), endoscopy (83%), CT scan (42.5%), and bone marrow biopsy (68%) (Supplementary Table S10). Peripheral blood smears were performed in 33 patients, with the most common findings being schistocytes (63.6%) and leukoerythroblasts (42.4%). Endoscopy was performed in 39 patients with a detailed description of findings available for 26 patients (Supplementary Table S11). Of these, upper endoscopy revealed ulcerations (57.7%), diffuse infiltration (30.8%), and a gastric mass (11.5%).

All patients had evidence of metastatic disease at the time of evaluation. The most common sites of metastases were bone (89.3%), bone marrow (68.1%), lymph nodes (48.9%), lung (23.4%), liver (19.1%), and lung (23.4%). CT (42.5%) and MRI (12.8%) scans were common staging modalities for staging.

### Treatment and Response

Information regarding treatment was available for 41 patients and are summarized in Table 2. Notably, 46.3% of patients did not receive any cancer-directed treatment. Cytotoxic chemotherapy (41.5%) was the most common in those who received treatment. Specific chemotherapeutic therapies that were used included 5-FU-based chemotherapy (94.4%) and cisplatin-based chemotherapy (72.2%). Details of the chemotherapy regimens used in our pooled cohort are provided in Supplementary Table S12. Most patients (57.1%) did not have any clinical or radiologic response despite treatment, the latter defined as reduction of tumor burden based on follow-up imaging.

**Table 2.** Summary of treatment and outcomes for pooled patient cohort.

	Pooled cohort	
	Number of patients	Results
Type of treatment, *n* (%)	41	
Chemotherapy alone		17 (41.5%)
Radiation		1 (2.4%)
Chemotherapy and radiation		4 (9.8%)
No treatment		19 (46.3%)
Follow-up time, median (IQR)	42	8 weeks (9.5 weeks)
Overall survival time, median (IQR)	40	8 weeks (10 weeks)
No treatment	18	4 weeks (9.3 weeks)
Treatment	19	10.3 weeks (4.4 weeks)
Survival outcome, *n* (%)	45	
Alive		4 (8.9%)
Death		41 (91.1%)
Cause of death, *n* (%)	18	
Hemorrhage related		11 (61.1%)
Respiratory failure		4 (22.2%)
Sepsis		1 (5.6%)
Carcinomatosis		2 (11.1%)

In terms of MAHA response, only 60% (9/15) of patients improved with therapy defined by amelioration in presenting anemia and/or thrombocytopenia. Notably, 78% (7/9) of patients remained transfusion-dependent even after treatment.

### Follow-up and Outcomes

Information with regards to survival outcomes was available for 45 patients. The median follow-up time was 8 weeks (range 0.1 to 76 weeks) after the presentation. Overall mortality was 91%. The overall cause of death was most commonly due to hemorrhage (61.1%), respiratory failure (22.2%), and carcinomatosis (11.1%).

### Survival Analysis

Next, we sought to understand the impact of MAHA on overall survival time by comparing our pooled cohort to patients with gastric SRCC with unknown MAHA status in a case-control analysis. From the publicly available SEER database, we identified a total of 6607 potential control patients with metastatic gastric SRCC with unknown MAHA status. Selection of our pooled cohort for the analysis excluded cases diagnosed before the year 2000 (11 cases) and those with missing follow-up time or outcome data (5 cases). Pooled cohort cases diagnosed before 2000 were excluded because patient statistics in the selected SEER database only started from 2000 onward. In a 1:4 matching ratio, we selected 124 SEER control patients, which were matched to the 31 cohort patients based on age, sex, and year of diagnosis. Given that all cases from the pooled cohort had metastatic disease, and control cases were selected from a cohort of metastatic gastric SRCC, these 2 groups were effectively matched for stage. Regression analysis was performed to identify characteristics that influenced survival. Covariates chosen from individual patient data from our systematic review and meta-data from SEER included age, sex, ethnicity, year of diagnosis, and follow-up time.

Baseline characteristics of the pooled cohort and SEER-derived control cohort are shown in Table 3. There were no significant differences in baseline parameters for both groups. After multivariate regression, we found that the presence of MAHA (hazard ratio [HR] 3.28, *P* < .01) and receiving no treatment (HR 3.57, *P* < .01) were associated with a significantly increased risk of mortality.

**Table 3.** Baseline characteristics of and multivariate regression analysis of selected patients from pooled patient cohort and SEER-matched controls for death and covariates.

Baseline characteristics	Pooled cohort (*n* = 31)	SEER control cohort (*n* = 124)	*P*-value*
Male sex, *n* (%)	18 (58.1%)	72 (58.1%)	
Age at diagnosis, *n* (%)			—
19-39	8 (25.8%)	32 (25.8%)	
40-59	11 (35.5%)	44 (35.5%)	
60+	12 (38.7%)	48 (38.7%)	
Year of diagnosis, *n* (%)			.84
2000-2009	10 (32.3%)	35 (28.2%)	
2010-2014	5 (16.1%)	25 (20.2%)	
2015-2020	16 (51.6%)	64 (51.6%)	
Race, *n* (%)			.19
Non-Hispanic White	15 (48.4%)	76 (61.3%)	
Non-White	16 (51.6%)	48 (38.7%)	
Any treatment, *n* (%)			.15
No	10 (32.3%)	26 (21%)	
Yes	20 (64.5%)	98 (79%)	
Unknown	1 (3.2%)	0 (0%)	
Treatment type, *n* (%)			.33
Chemotherapy	14 (45.2%)	64 (51.6%)	
Other treatment	6 (19.4%)	34 (27.4%)	
No treatment	10 (32.3%)	26 (21.0%)	
Unknown	1 (3.2%)	0 (0%)	

Regression analysis	Hazard ratio	95% CI of HR	*P*-value
Mortality			
SEER-matched cohort	Reference	Reference	<.01
Pooled study cohort	3.28	2.11-5.12	
Age at diagnosis			
19-39	Reference	Reference	
40-59	0.75	0.47-1.20	.23
60+	0.86	0.53-1.39	.54
Sex			
Male	Reference	Reference	
Female	0.93	0.65-1.34	.69
Race			
Non-Hispanic White	Reference	Reference	
Non-White	0.82	0.55-1.22	.32
Year of diagnosis			
2000-2009	Reference	Reference	Reference
2010-2014	0.75	0.45-1.24	.26
2015-2020	0.56	0.37-0.86	.01
Treatment			
Chemotherapy	Reference	Reference	Reference
Other treatment	1.02	0.67-1.55	.95
No treatment	3.57	2.30-5.56	<.01

*P-values are based on a Pearson’s *χ*^2^ test for categorical variables.

**Figure 4.**
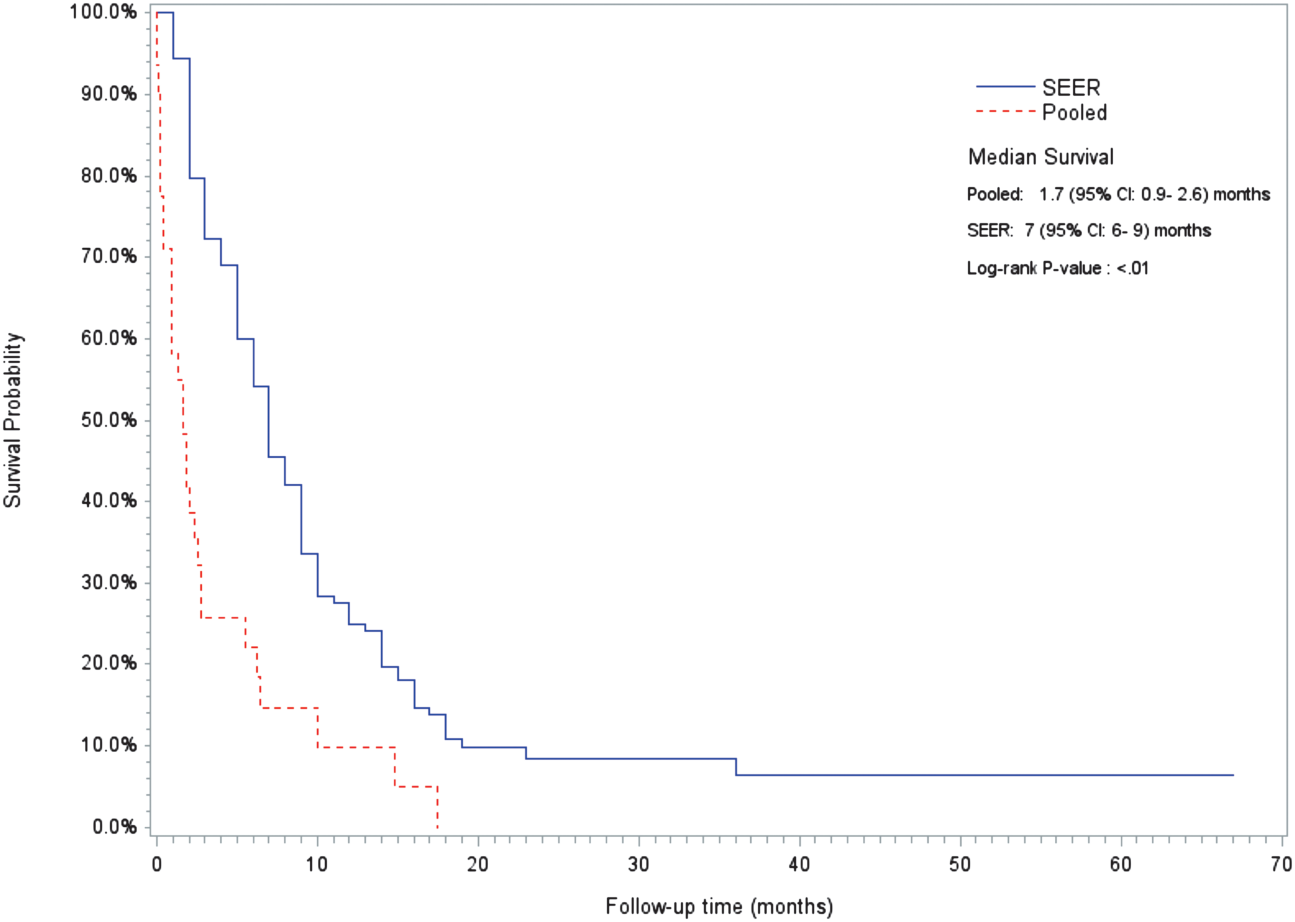
Kaplan-Meier survival curves for SEER-derived metastatic gastric SRCC and pooled cohort of metastatic gastric SRCC complicated by MAHA.

Kaplan-Meier survival curves for metastatic gastric SRCC and metastatic gastric SRCC complicated by MAHA is shown in Fig. 4. Survival in the MAHA cohort was significantly less than that of the SEER cohort (7 vs 28 weeks; *P* < .01).

## Discussion

Our study highlights MAHA as a rare, late-stage complication of gastric SRCC. MAHA is often the first overt manifestation of metastatic SRCC. In fact, all patients in our analysis had metastatic disease at index evaluation, commonly to the bone, and lymph nodes. Endoscopy, bone marrow biopsy, and CT imaging are useful diagnostic studies to identify the origin of the primary cancer. Even with treatment, the overall prognosis is poor, with higher mortality rates and worse survival than patients with metastatic gastric SRCC without MAHA.

Cancer-related MAHA (CR-MAHA) is a type of Coombs-negative thrombotic microangiopathy observed in solid cancers and is characterized by erythrocyte fragmentation. Evaluation will reveal schistocytes on blood smear, elevated LDH, low or absent haptoglobin, thrombocytopenia, and elevated total bilirubin levels.^17^ Schistocytes are fragments of damaged red blood cells formed from the excessive wall shear stress on red blood cells in MAHA; they can be visualized on blood smear as helmet cells or irregular, triangular, and crescent-shaped cells lacking central pallor.^18^

Among CR-MAHA, gastric cancer is the most common cancer type, and adenocarcinomas are the most common histologic type.^7^ The reason for this is unknown. Signet ring cell carcinoma comprises 16.8% of all gastric cancer cases and has a strong predilection for gastric tissue.^19^ While the pathogenesis of MAHA and gastric SRCC remains unclear, several mechanisms are postulated to explain their association. Signet ring cell carcinoma is an adenocarcinoma that produces mucin, which has erythrocytopathic activity.^20^ Moreover, mucinous tumors secrete enzymes that can activate factor X and provoke DIC.^21^ In patients with solid neoplasms like gastric cancer, hemolysis primarily occurs from the mechanical shearing of red blood cells passing through small blood vessels, especially in infiltrated organs such as the bone marrow.^22,23^ Direct invasion of the endothelium may also lead to cell injury, triggering thrombotic microangiopathies.^24^ Finally, tumor cells can produce cytokines which activate tumor necrosis factor-mediated red cell damage.^25^

Our case-control analysis using SEER data showed that patients with metastatic gastric SRCC and MAHA have worse survival than age and sex-matched control patients with metastatic gastric SRCC (MAHA status unknown). We propose that MAHA is a surrogate marker for metastatic disease, primarily responsible for serious, fatal outcomes. It is well established that patients with diffuse metastatic gastric adenocarcinoma, including the SRCC type, have a poor prognosis.^26-28^ Of note, all our patients had metastatic disease at initial presentation. Increased risk of mortality and short survival time is common in disseminated gastric SRCC and MAHA cases. Of note, MAHA associated with cancers of other primary sites also show very similar poor outcomes. In one of the largest reviews of CR-MAHA, survival was poor in cancers originating from the breast (median 0.5 months without chemotherapy/surgery, 4 months with chemotherapy/surgery), lung (median 0.5 months without chemotherapy/surgery, 5 months with chemotherapy/surgery), and unknown primary (median 0.5 months without chemotherapy/surgery, 3.5 months with chemotherapy/surgery).^7^ Plasma exchange and fresh frozen plasma was rarely effective in patients with gastric, breast, lung or unknown primary cancers associated with MAHA. Nearly all the patients in that study also had metastatic disease, further suggesting that metastatic disease as the factor responsible for the demise of affected patients.

Palliative treatments exist for both metastatic SRCC and MAHA, although simultaneous management of both conditions can be competing and challenging. Treatment for metastatic SRCC includes chemotherapy and radiation.^26^ Chemotherapy should be started as soon as possible.^29^ Blood products transfusion is often required.^22^ Combination chemotherapy has been well-established in the palliative treatment of advanced-stage gastric cancer and includes fluoropyrimidine (5FU or capecitabine) and oxaliplatin, fluoropyrimidine (5FU or capecitabine) and cisplatin; for patients with HER2 positive tumors, adding trastuzumab is recommended and for patients with tumors that are CPS > 5, adding nivolumab is recommended.^30^ In our cohort of patients, most had chemotherapy regimens consisting of 5-flurouracil and cisplatin. TEFOX (docetaxel-5FU-oxaliplatin) has been evaluated as an alternative option for SRCC in a recent retrospective study.^31^ However, prognosis remains poor due to extensive metastatic disease, short remission, and complications from MAHA (like hemorrhage) which can be life-threatening. In fact, for many patients, they may already be too ill and lack the clinical stability to initiate chemotherapy.

The clinical significance of our study lies in highlighting the most common clinical phenotype of a patient with gastric SRCC and MAHA at presentation. Our study also underscores the inisiduous nature of the disease process given that MAHA was present when the initial diagnosis of gastric SRCC was made for nearly all the patients in our cohort. If a patient presents with back and bone pain along with evidence of hemolytic anemia, advanced stage gastric SRCC with MAHA should be considered on the differential and an EGD can be considered. Similarly, a finding of MAHA in should prompt the clinician to rule out gastric cancer in patients at risk. If the latter is found with MAHA, thorough cross-sectional imaging needs to be pursued given this association between MAHA and distant metastases. Additionally, the summary of our study findings can provide prognostic information to patients and families. It should be emphasized that prognosis is potentially poor and aggressive therapy may not yield significant survival benefits. A multidisciplinary discussion which involves gastroenterology, oncology, and palliative care medicine is thus warranted.

A key strength of our study is the systematic methodology used to capture all gastric SRCC and MAHA cases in the literature to describe the clinical presentation, evaluation, and outcomes of this rare phenotype. We also used the large publicly available SEER database to identify stage, age, and sex-matched controls and highlight differences in metastatic gastric SRCC survival with and without MAHA. Another strength of our study was in implelemting a novel methodology of combining a case study, systematic review, and case-control analysis approach.^16,32^ A systematic review alone may be insufficient to provide guidance in informing decisions in care.^13^ Combining health system data with a systematic review approach can further strengthen the evidence and enhance the applicability of systematic review findings.^13^ In our study, the systematic review provided a comprehensive context to understand the patient’s overall disease course and medical management in the case study. Similarly, the case study and case-control analysis were complimentary to the systematic review by expanding the evidence base given the limited number of total cases in the literature.

Our study has several inherent limitations that should be acknowledged. Several data parameters were missing and reporting bias is likely present. Importantly, selection bias is likely present given that individual patient data was derived from case reports and case series. However, evidence from case reports and series takes on increased significance when higher levels of evidence are not available.^14^ In addition, given patients with gastric SRCC and MAHA have short survival time with high rates of mortality, patients may have perished prior to work-up and were thus not reported. Moreover, an era effect is likely present given the inclusion of data published between 1985 to 2020, with 2011 as the median year of publication. Since there have been advances over time regarding the diagnostic and therapeutic approaches, earlier study findings may not be generalizable for patients in the current day and age. However, our case study survival outcome was similar to that of the overall cohort even when cases diagnosed before 2000 were excluded for the case-control analysis. Lastly, it is important to note that the MAHA status within our SEER cohort was unknown. However, a significantly longer survival time of SEER cohort compared with our MAHA-proven cohort was demonstrated, despite matching of other important parameters.

## Conclusion

In conclusion, MAHA is a late-stage complication of gastric SRCC that is a surrogate marker for metastatic disease. Cancer-directed treatment and treatment for MAHA both appear to provide minimal benefit. Overall prognosis is poor with short survival despite treatment.

## Supplementary Material

oyac093_suppl_Supplementary_Material

## Data Availability

The data underlying this article will be shared on reasonable request to the corresponding author.

## References

[1] Venerito M , Vasapolli R, Rokkas T, et al. Gastric cancer: epidemiology, prevention, and therapy. Helicobacter. 2018;23(Suppl 1):e12518e12518. https://doi.org/10.1111/hel.12518. 30203589 10.1111/hel.12518

[2] Nagtegaal ID , Odze RD, Klimstra D, et al. The 2019 who classification of tumours of the digestive system. Histopathology. 2020;76(2):182-188. https://doi.org/10.1111/his.13975. 31433515 10.1111/his.13975 PMC7003895

[3] Abdel Samie A , Sandritter B, Theilmann L. Severe microangiopathic hemolytic anemia as first manifestation of a cup syndrome. Rapid hematologic remission under polychemotherapy. Med Klin. 2004;99(3):148-153. https://doi.org/10.1007/s00063-004-1023-8. 10.1007/s00063-004-1023-8 15024487

[4] Henson DE , Dittus C, Younes M, et al. Differential trends in the intestinal and diffuse types of gastric carcinoma in the united states, 1973-2000: Increase in the signet ring cell type. Arch Pathol Lab Med. 2004;128(7):765-770. https://doi.org/10.5858/2004-128-765-DTITIA. 15214826 10.5858/2004-128-765-DTITIA

[5] Nitsche U , Zimmermann A, Späth C, et al. Mucinous and signet-ring cell colorectal cancers differ from classical adenocarcinomas in tumor biology and prognosis. Ann Surg 2013;258(5):775-82; discussion 782-773. https://doi.org/10.1097/SLA.0b013e3182a69f7e 23989057 10.1097/SLA.0b013e3182a69f7e PMC3888475

[6] Pernot S , Voron T, Perkins G, et al. Signet-ring cell carcinoma of the stomach: impact on prognosis and specific therapeutic challenge. World J Gastroenterol. 2015;21(40):11428-11438. https://doi.org/10.3748/wjg.v21.i40.11428. 26523107 10.3748/wjg.v21.i40.11428 PMC4616218

[7] Lechner K , Obermeier HL. Cancer-related microangiopathic hemolytic anemia: clinical and laboratory features in 168 reported cases. Medicine (Baltimore). 2012;91(4):195-205. https://doi.org/10.1097/MD.0b013e3182603598. 22732949 10.1097/MD.0b013e3182603598

[8] Brain MC , Dacie JV, Hourihane DO. Microangiopathic haemolytic anaemia: the possible role of vascular lesions in pathogenesis. Br J Haematol. 1962;8:358-374. https://doi.org/10.1111/j.1365-2141.1962.tb06541.x. 14014893 10.1111/j.1365-2141.1962.tb06541.x

[9] Mayer SA , Aledort LM. Thrombotic microangiopathy: differential diagnosis, pathophysiology and therapeutic strategies. Mt Sinai J Med. 2005;72(3):166-175. 15915311

[10] Riley DS , Barber MS, Kienle GS, et al. Care guidelines for case reports: explanation and elaboration document. J Clin Epidemiol. 2017;89:218-235. https://doi.org/10.1016/j.jclinepi.2017.04.026. 28529185 10.1016/j.jclinepi.2017.04.026

[11] Page MJ , McKenzie JE, Bossuyt PM, et al. The prisma 2020 statement: an updated guideline for reporting systematic reviews. BMJ. 2021;372:n71. https://doi.org/10.1136/bmj.n71. 33782057 10.1136/bmj.n71 PMC8005924

[12] Campbell M , McKenzie JE, Sowden A, et al. Synthesis without meta-analysis (swim) in systematic reviews: reporting guideline. BMJ. 2020;368:l6890. https://doi.org/10.1136/bmj.l6890. 31948937 10.1136/bmj.l6890 PMC7190266

[13] Lin JS , Murad MH, Leas B, et al. A narrative review and proposed framework for using health system data with systematic reviews to support decision-making. J Gen Intern Med. 2020;35(6):1830-1835. https://doi.org/10.1007/s11606-020-05783-5. 32239462 10.1007/s11606-020-05783-5 PMC7280421

[14] Murad MH , Sultan S, Haffar S, et al. Methodological quality and synthesis of case series and case reports. BMJ Evid Based Med. 2018;23(2):60-63. https://doi.org/10.1136/bmjebm-2017-110853. 10.1136/bmjebm-2017-110853 PMC6234235 29420178

[15] Bazerbachi F , Haffar S, Sugihara T, et al. Peribiliary cysts: a systematic review and proposal of a classification framework. BMJ Open Gastroenterol. 2018;5(1):e000204. https://doi.org/10.1136/bmjgast-2018-000204. 10.1136/bmjgast-2018-000204 PMC6001913 29915665

[16] Li DK , Haffar S, Horibe M, et al. Verrucous esophageal carcinoma is a unique indolent subtype of squamous cell carcinoma: a systematic review and individual patient regression analysis. J Gastroenterol. 2021;56(1):12-24. https://doi.org/10.1007/s00535-020-01736-1. 33079233 10.1007/s00535-020-01736-1

[17] Donato AA , Nazir S, Tachamo N, et al. Cancer-related microangiopathic haemolytic anaemia. BMJ Case Rep. 2017;2017:bcr-2017-bc223382. 10.1136/bcr-2017-223382 PMC5720307 29183903

[18] Zini G , d’Onofrio G, Briggs C, et al. Icsh recommendations for identification, diagnostic value, and quantitation of schistocytes. Int J Lab Hematol. 2012;34(2):107-116. https://doi.org/10.1111/j.1751-553X.2011.01380.x. 22081912 10.1111/j.1751-553X.2011.01380.x

[19] Benesch MGK , Mathieson A. Epidemiology of signet ring cell adenocarcinomas. Cancers (Basel). 2020;12(6):1-34. 10.3390/cancers12061544 PMC7352645 32545410

[20] Gaspar BL , Sharma P, Das R. Anemia in malignancies: pathogenetic and diagnostic considerations. Hematology. 2015;20(1):18-25. https://doi.org/10.1179/1607845414Y.0000000161. 24666207 10.1179/1607845414Y.0000000161

[21] Morton JM , George JN. Microangiopathic hemolytic anemia and thrombocytopenia in patients with cancer. J Oncol Pract. 2016;12(16):523-530. https://doi.org/10.1200/JOP.2016.012096. 27288467 10.1200/JOP.2016.012096

[22] Thomas MR , Scully M. How i treat microangiopathic hemolytic anemia in patients with cancer. Blood. 2021;137(10):1310-1317. https://doi.org/10.1182/blood.2019003810. 33512445 10.1182/blood.2019003810 PMC8555418

[23] Govind Babu K , Bhat GR. Cancer-associated thrombotic microangiopathy. Ecancermedicalscience. 2016;10:649-649. https://doi.org/10.3332/ecancer.2016.649. 27433282 10.3332/ecancer.2016.649 PMC4929977

[24] Goldberg RJ , Nakagawa T, Johnson RJ, et al. The role of endothelial cell injury in thrombotic microangiopathy. Am J Kidney Dis. 2010;56(6):1168-1174. https://doi.org/10.1053/j.ajkd.2010.06.006. 20843591 10.1053/j.ajkd.2010.06.006 PMC3148799

[25] Pendse AA , Edgerly CH, Fedoriw Y. Hemolytic anemia and metastatic carcinoma: case report and literature review. Lab Med. 2014;45(2):132-135. https://doi.org/10.1309/lm6fenwrxx5grwbt. 24868993 10.1309/lm6fenwrxx5grwbt

[26] Machlowska J , Pucułek M, Sitarz M, et al. State of the art for gastric signet ring cell carcinoma: from classification, prognosis, and genomic characteristics to specified treatments. Cancer Manag Res. 2019;11:2151-2161. https://doi.org/10.2147/CMAR.S188622. 30936747 10.2147/CMAR.S188622 PMC6421895

[27] Brenner H , Rothenbacher D, Arndt V. Epidemiology of stomach cancer. Methods Mol Biol. 2009;472:467-477. https://doi.org/10.1007/978-1-60327-492-0_23. 19107449 10.1007/978-1-60327-492-0_23

[28] Yang D , Hendifar A, Lenz C, et al. Survival of metastatic gastric cancer: significance of age, sex and race/ethnicity. J Gastrointest Oncol. 2011;2(2):77-84. https://doi.org/10.3978/j.issn.2078-6891.2010.025. 22811834 10.3978/j.issn.2078-6891.2010.025 PMC3397601

[29] Shi T , Huang M, Han D, et al. Chemotherapy is associated with increased survival from colorectal signet ring cell carcinoma with distant metastasis: a surveillance, epidemiology, and end results database analysis. Cancer Med. 2019;8(4):1930-1940. https://doi.org/10.1002/cam4.2054. 30864303 10.1002/cam4.2054 PMC6488115

[30] Network NCC. Gastric cancer (version 1.2022). Accessed January 10, 2022. https://www.nccn.org/professionals/physician_gls/pdf/gastric.pdf.

[31] Pernot S , Dubreuil O, Aparicio T, et al. Efficacy of a docetaxel-5fu-oxaliplatin regimen (tefox) in first-line treatment of advanced gastric signet ring cell carcinoma: an ageo multicentre study. Br J Cancer. 2018;119(4):424-428. https://doi.org/10.1038/s41416-018-0133-7. 29872148 10.1038/s41416-018-0133-7 PMC6133962

[32] Bazerbachi F , Haffar S, Szarka LA, et al. Secretory diarrhea and hypokalemia associated with colonic pseudo-obstruction: a case study and systematic analysis of the literature. Neurogastroenterol Motil. 2017;29(11):e13120. https://doi.org/10.1111/nmo.13120. 10.1111/nmo.13120 28580600

