# Supplementary material for: Microangiopathic Hemolytic Anemia Is a Late and Fatal Complication of Gastric Signet Ring Cell Carcinoma: A Systematic Review and Case-Control Study
Source: Oncologist. 2022 May 19;27(9):751–9. doi: 10.1093/oncolo/oyac093 (PMC9438916; doi:10.1093/oncolo/oyac093)
Supplement: oyac093_suppl_Supplementary_Material [file oyac093_suppl_supplementary_material.docx]

**Supplemental Table 1. PRISMA Reporting Guideline Checklists**

| **Topic** | **No.** | **Item** | **Location where item is reported** |
| --- | --- | --- | --- |
| **TITLE** |  |  |  |
| **Title** | 1 | Identify the report as a systematic review. | LN1-2 |
| **ABSTRACT** |  |  |  |
| **Abstract** | 2 | See the PRISMA 2020 for Abstracts checklist | Suppl. 2 |
| **INTRODUCTION** |  |  |  |
| **Rationale** | 3 | Describe the rationale for the review in the context of existing knowledge. | LN87-97 |
| **Objectives** | 4 | Provide an explicit statement of the objective(s) or question(s) the review addresses. | LN99-105 |
| **METHODS** |  |  |  |
| **Eligibility criteria** | 5 | Specify the inclusion and exclusion criteria for the review and how studies were grouped for the syntheses. | LN171-174 |
| **Information sources** | 6 | Specify all databases, registers, websites, organisations, reference lists and other sources searched or consulted to identify studies. Specify the date when each source was last searched or consulted. | LN166-170 |
| **Search strategy** | 7 | Present the full search strategies for all databases, registers and websites, including any filters and limits used. | Suppl. 4 |
| **Selection process** | 8 | Specify the methods used to decide whether a study met the inclusion criteria of the review, including how many reviewers screened each record and each report retrieved, whether they worked independently, and if applicable, details of automation tools used in the process. | LN180-184, LN197-198 |
| **Data collection process** | 9 | Specify the methods used to collect data from reports, including how many reviewers collected data from each report, whether they worked independently, any processes for obtaining or confirming data from study investigators, and if applicable, details of automation tools used in the process. | LN180-184 |
| **Data items** | 10a | List and define all outcomes for which data were sought. Specify whether all results that were compatible with each outcome domain in each study were sought (e.g. for all measures, time points, analyses), and if not, the methods used to decide which results to collect. | LN184-188 |
|  | 10b | List and define all other variables for which data were sought (e.g. participant and intervention characteristics, funding sources). Describe any assumptions made about any missing or unclear information. | LN205-208 |
| **Study risk of bias assessment** | 11 | Specify the methods used to assess risk of bias in the included studies, including details of the tool(s) used, how many reviewers assessed each study and whether they worked independently, and if applicable, details of automation tools used in the process. | LN178-180 |
| **Effect measures** | 12 | Specify for each outcome the effect measure(s) (e.g. risk ratio, mean difference) used in the synthesis or presentation of results. | LN216-223 |
| **Synthesis methods** | 13a | Describe the processes used to decide which studies were eligible for each synthesis (e.g. tabulating the study intervention characteristics and comparing against the planned groups for each synthesis (item 5)). | LN171-174 |
|  | 13b | Describe any methods required to prepare the data for presentation or synthesis, such as handling of missing summary statistics, or data conversions. | LN180-188 |
|  | 13c | Describe any methods used to tabulate or visually display results of individual studies and syntheses. | LN216-223 |
|  | 13d | Describe any methods used to synthesize results and provide a rationale for the choice(s). If meta-analysis was performed, describe the model(s), method(s) to identify the presence and extent of statistical heterogeneity, and software package(s) used. | LN210-223 |
|  | 13e | Describe any methods used to explore possible causes of heterogeneity among study results (e.g. subgroup analysis, meta-regression). | LN210-223 |
|  | 13f | Describe any sensitivity analyses conducted to assess robustness of the synthesized results. | LN210-223 |
| **Reporting bias assessment** | 14 | Describe any methods used to assess risk of bias due to missing results in a synthesis (arising from reporting biases). | LN178-180 |
| **Certainty assessment** | 15 | Describe any methods used to assess certainty (or confidence) in the body of evidence for an outcome. | LN220-223 |
| **RESULTS** |  |  |  |
| **Study selection** | 16a | Describe the results of the search and selection process, from the number of records identified in the search to the number of studies included in the review, ideally using a flow diagram. | LN226-229 |
|  | 16b | Cite studies that might appear to meet the inclusion criteria, but which were excluded, and explain why they were excluded. | Suppl. 5 |
| **Study characteristics** | 17 | Cite each included study and present its characteristics. | LN226-229 |
| **Risk of bias in studies** | 18 | Present assessments of risk of bias for each included study. | LN232-234 |
| **Results of individual studies** | 19 | For all outcomes, present, for each study: (a) summary statistics for each group (where appropriate) and (b) an effect estimate and its precision (e.g. confidence/credible interval), ideally using structured tables or plots. | LN237-280 |
| **Results of syntheses** | 20a | For each synthesis, briefly summarise the characteristics and risk of bias among contributing studies. | LN283-305 |
|  | 20b | Present results of all statistical syntheses conducted. If meta-analysis was done, present for each the summary estimate and its precision (e.g. confidence/credible interval) and measures of statistical heterogeneity. If comparing groups, describe the direction of the effect. | LN283-305 |
|  | 20c | Present results of all investigations of possible causes of heterogeneity among study results. | LN283-305 |
|  | 20d | Present results of all sensitivity analyses conducted to assess the robustness of the synthesized results. | LN283-305 |
| **Reporting biases** | 21 | Present assessments of risk of bias due to missing results (arising from reporting biases) for each synthesis assessed. | LN232-234 |
| **Certainty of evidence** | 22 | Present assessments of certainty (or confidence) in the body of evidence for each outcome assessed. | LN299-305 |
| **DISCUSSION** |  |  |  |
| **Discussion** | 23a | Provide a general interpretation of the results in the context of other evidence. | LN307-376 |
|  | 23b | Discuss any limitations of the evidence included in the review. | LN394-395 |
|  | 23c | Discuss any limitations of the review processes used. | LN392-403 |
|  | 23d | Discuss implications of the results for practice, policy, and future research. | LN406-408 |
| **OTHER INFORMATION** |  |  |  |
| **Registration and protocol** | 24a | Provide registration information for the review, including register name and registration number, or state that the review was not registered. | LN159 |
|  | 24b | Indicate where the review protocol can be accessed, or state that a protocol was not prepared. | LN159 |
|  | 24c | Describe and explain any amendments to information provided at registration or in the protocol. | N/A |
| **Support** | 25 | Describe sources of financial or non-financial support for the review, and the role of the funders or sponsors in the review. | LN40 |
| **Competing interests** | 26 | Declare any competing interests of review authors. | LN42-45 |
| **Availability of data, code and other materials** | 27 | Report which of the following are publicly available and where they can be found: template data collection forms; data extracted from included studies; data used for all analyses; analytic code; any other materials used in the review. | LN |

From: Page MJ, McKenzie JE, Bossuyt PM, Boutron I, Hoffmann TC, Mulrow CD, et al. The PRISMA 2020 statement: an updated guideline for reporting systematic reviews. MetaArXiv. 2020, September 14. DOI: 10.31222/osf.io/v7gm2. For more information, visit: www.prisma-statement.org

# Supplemental Table 2. PRIMSA Abstract Checklist

| **Topic** | **No.** | **Item** | **Reported?** |
| --- | --- | --- | --- |
| **TITLE** |  |  |  |
| **Title** | 1 | Identify the report as a systematic review. | Yes |
| **BACKGROUND** |  |  |  |
| **Objectives** | 2 | Provide an explicit statement of the main objective(s) or question(s) the review addresses. | Yes |
| **METHODS** |  |  |  |
| **Eligibility criteria** | 3 | Specify the inclusion and exclusion criteria for the review. | Yes |
| **Information sources** | 4 | Specify the information sources (e.g. databases, registers) used to identify studies and the date when each was last searched. | Yes |
| **Risk of bias** | 5 | Specify the methods used to assess risk of bias in the included studies. | Yes |
| **Synthesis of results** | 6 | Specify the methods used to present and synthesize results. | Yes |
| **RESULTS** |  |  |  |
| **Included studies** | 7 | Give the total number of included studies and participants and summarise relevant characteristics of studies. | Yes |
| **Synthesis of results** | 8 | Present results for main outcomes, preferably indicating the number of included studies and participants for each. If meta-analysis was done, report the summary estimate and confidence/credible interval. If comparing groups, indicate the direction of the effect (i.e. which group is favoured). | Yes |
| **DISCUSSION** |  |  |  |
| **Limitations of evidence** | 9 | Provide a brief summary of the limitations of the evidence included in the review (e.g. study risk of bias, inconsistency and imprecision). | Yes |
| **Interpretation** | 10 | Provide a general interpretation of the results and important implications. | Yes |
| **OTHER** |  |  |  |
| **Funding** | 11 | Specify the primary source of funding for the review. | - |
| **Registration** | 12 | Provide the register name and registration number. | - |

**Supplementary Table 3. Synthesis Without Meta-analysis (SWiM) explanation and elaboration checklist**

| **SWiM reporting item** | **Item description** | **Page in manuscript where item is reported** | **Other*** |
| --- | --- | --- | --- |
| *Methods* | | | |
| **1** Grouping studies for synthesis | 1a) Provide a description of, and rationale for, the groups used in the synthesis (e.g., groupings of populations, interventions, outcomes, study design) | 9-10 |  |
|  | 1b) Detail and provide rationale for any changes made subsequent to the protocol in the groups used in the synthesis | N/A |  |
| **2** Describe the standardised metric and transformation methods used | Describe the standardised metric for each outcome. Explain why the metric(s) was chosen, and describe any methods used to transform the intervention effects, as reported in the study, to the standardised metric, citing any methodological guidance consulted | 10 |  |
| **3** Describe the synthesis methods | Describe and justify the methods used to synthesise the effects for each outcome when it was not possible to undertake a meta-analysis of effect estimates | 9-10 |  |
| **4** Criteria used to prioritise results for summary and synthesis | Where applicable, provide the criteria used, with supporting justification, to select the particular studies, or a particular study, for the main synthesis or to draw conclusions from the synthesis (e.g., based on study design, risk of bias assessments, directness in relation to the review question) | 9-10 |  |
| **5** Investigation of heterogeneity in reported effects | State the method(s) used to examine heterogeneity in reported effects when it was not possible to undertake a meta-analysis of effect estimates and its extensions to investigate heterogeneity | 9-10 |  |
| **6** Certainty of evidence | Describe the methods used to assess certainty of the synthesis findings | 10 |  |
| **7** Data presentation methods | Describe the graphical and tabular methods used to present the effects (e.g., tables, forest plots, harvest plots).  Specify key study characteristics (e.g., study design, risk of bias) used to order the studies, in the text and any tables or graphs, clearly referencing the studies included | Figure 4, Supplementary 8 |  |
| *Results* | | | |
| **8** Reporting results | For each comparison and outcome, provide a description of the synthesised findings, and the certainty of the findings. Describe the result in language that is consistent with the question the synthesis addresses, and indicate which studies contribute to the synthesis | 13-14, Table 1 and 2 |  |
| *Discussion* |  |  |  |
| **9** Limitations of the synthesis | Report the limitations of the synthesis methods used and/or the groupings used in the synthesis, and how these affect the conclusions that can be drawn in relation to the original review question | 18 |  |

PRISMA=Preferred Reporting Items for Systematic Reviews and Meta-Analyses.

*If the information is not provided in the systematic review, give details of where this information is available (e.g., protocol, other published papers (provide citation details), or website (provide the URL)).

**Supplementary Table 4. Search Strategies**

| **Ovid Embase**  1. signet ring carcinoma/  2. ((signet* or castration cell*) adj3 (adenocarcinoma* or carcinoma* or malignan* or tumor* or tumour* or cancer*)).tw,kw.  3. 1 or 2  4. thrombotic thrombocytopenic purpura/  5. ((Moschcowitz or moschowitz) adj3 (disease* or syndrome* or disorder*)).tw,kw.  6. (thrombotic adj3 (thrombocytopenia* or thrombocytopaenia* or microangiopath* or purpura*)).tw,kw.  7. (essential* adj3 (thrombocytopaenia* or thrombocytopenia*)).tw,kw.  8. (microangiopathic adj3 (haemolytic or hemolytic or anaemia* or anemia*)).tw,kw.  9. (schulman adj2 upshaw).tw,kw.  10. 4 or 5 or 6 or 7 or 8 or 9  11. 3 and 10 |
| --- |
| **Ovid MEDLINE(R) ALL**  1. exp Carcinoma, Signet Ring Cell/  2. ((signet* or castration cell*) adj3 (adenocarcinoma* or carcinoma* or malignan* or tumor* or tumour* or cancer*)).tw,kw.  3. 1 or 2  4. Purpura, Thrombotic Thrombocytopenic /  5. ((Moschcowitz or moschowitz) adj3 (disease* or syndrome* or disorder*)).tw,kw.  6. (thrombotic adj3 (thrombocytopenia* or thrombocytopaenia* or microangiopath* or purpura*)).tw,kw.  7. (essential* adj3 (thrombocytopaenia* or thrombocytopenia*)).tw,kw.  8. (microangiopathic adj3 (haemolytic or hemolytic or anaemia* or anemia*)).tw,kw.  9. (schulman adj2 upshaw).tw,kw.  10. 4 or 5 or 6 or 7 or 8 or 9  11. 3 and 10 |
| **Scopus**  ( TITLE-ABS-KEY ( ( signet* OR "castration cell*" ) W/3 ( adenocarcinoma* OR carcinoma* OR malignan* OR tumor* OR tumour* OR cancer* ) ) ) AND ( TITLE-ABS-KEY ( ( moschcowitz OR moschowitz ) W/3 ( disease* OR syndrome* OR disorder* ) ) OR TITLE-ABS-KEY ( thrombotic W/3 ( thrombocytopenia* OR thrombocytopaenia* OR microangiopath* OR purpura* ) ) OR TITLE-ABS-KEY ( essential* W/3 ( thrombocytopaenia* OR thrombocytopenia* ) ) OR TITLE-ABS-KEY ( microangiopathic W/3 ( haemolytic OR hemolytic OR anaemia* OR anemia* ) ) OR TITLE-ABS-KEY ( schulman W/2 upshaw ) ) |
| **Web of Science Core Collection**  #1 TS= ((signet* OR "castration cell*" ) near/3 ( adenocarcinoma* OR carcinoma* OR malignan* OR tumor* OR tumour* OR cancer* ) )  #2 TS=((moschcowitz OR moschowitz ) near/3 ( disease* OR syndrome* OR disorder* ) ) OR TS= ( thrombotic near/3 ( thrombocytopenia* OR thrombocytopaenia* OR microangiopath* OR purpura* ) ) OR TS=( essential* near/3 ( thrombocytopaenia* OR thrombocytopenia* ) ) OR TS=( microangiopathic near/3 ( haemolytic OR hemolytic OR anaemia* OR anemia* ) ) OR TS=( schulman near/2 upshaw )  #3 #1 and #2 |
| **Pubmed**  (moschcowitz disease* OR moschowitz disease* or moschcowitz syndrome* OR moschowitz syndrome* or moschcowitz disorder* OR moschowitz disorder* or thrombotic thrombocytopenia* OR thrombotic thrombocytopaenia* OR thrombotic microangiopath* OR thrombotic purpura* or essential* thrombocytopaenia* OR essential* thrombocytopenia* or microangiopathic haemolytic OR microangiopathic hemolytic OR microangiopathic anaemia* OR microangiopathic anemia* or schulman upshaw) AND (signet* adenocarcinoma* OR "castration cell*" adenocarcinoma* or signet* carcinoma* OR "castration cell*" carcinoma* or signet* malignan* OR "castration cell*" malignan* or signet* tumor* OR "castration cell*" tumor* or signet* tumour* OR "castration cell*" tumour* or signet* cancer* OR "castration cell*"cancer*) |
| **Cochrane Library**  #1 ((signet* OR "castration cell*" ) near/3 ( adenocarcinoma* OR carcinoma* OR malignan* OR tumor* OR tumour* OR cancer* ) ):ti,ab  #2 ((moschcowitz OR moschowitz ) near/3 ( disease* OR syndrome* OR disorder*)):ti,ab OR (thrombotic near/3 (thrombocytopenia* OR thrombocytopaenia* OR microangiopath* OR purpura*)):ti,ab OR (essential* near/3 (thrombocytopaenia* OR thrombocytopenia*)):ti,ab OR (microangiopathic near/3 (haemolytic OR hemolytic OR anaemia* OR anemia*)):ti,ab OR (schulman near/2 upshaw):ti,ab  #3 #1 and #2 |
| **Japan Medical Abstracts Society**  #1 (印鑑細胞癌/AL or 印環細胞癌/TH) and (PT=血小板減少症 and PT=紫斑病) |
| **Google Scholar**  Signet cell microangiopathic anemia |

**Supplemental Table 5. Excluded Studies**

| **First Authors Last Name** | **Year** | **Title** | **Journal** | **Reason for Exclusion** |
| --- | --- | --- | --- | --- |
| Almahroos | 2016 | Signet-ring gastric carcinoma, at Bahrain | Acta Medica International | No MAHA described |
| Arslan | 2014 | Her-2 positive gastric cancer presented with thrombocytopenia and skin involvement: a case report | Case reports in oncological medicine | No MAHA described |
| Bucaloiu | 2003 | Thrombotic thrombocytopenic purpura and extensive bone marrow necrosis secondary to metastatic signet ring cell adenocarcinoma | Journal of General Internal Medicine | Insufficient patient information |
| Butler | 2014 | Isolated hemolytic anemia: An unusual manifestation of occult malignancy | Hematology Reports | Wrong patient population |
| Choy | 2016 | Cancer-related microangiopathic hemolytic anemia | Transfusion | Insufficient patient information |
| Dempke | 2000 | [Hemorrhagic diathesis as initial symptom of stomach carcinoma] | Wiener klinische Wochenschrift | Duplicate study data |
| Eisa | 2018 | A Metastatic Signet Ring Cell Carcinoma Presented as Acquired Thrombotic Thrombocytopenic Purpura: A Case Report | Journal of Hematology | Wrong patient population |
| Elman | 2014 | Report of rare urachal carcinoma presenting with thrombocytopenia and microangiopathic hemolytic anemia | Blood. Conference: 56th Annual Meeting of the American Society of Hematology, ASH | Wrong patient population |
| Francis | 2007 | Disseminated malignancy misdiagnosed as thrombotic thrombocytopenic purpura: a report of 10 patients and a systematic review of published cases | The Oncologist | Wrong patient population |
| Gudipally | 2019 | 2732 Signet Ring Cell Carcinoma: A Rare Case Report Masquerading as Digital Infarction | Official journal of the American College of Gastroenterology\| ACG | Insufficient patient information |
| Guner | 2015 | Microangiopathic Hemolytic Anemia (MAHA), High Alkaline Phosphatase and D-dimer Levels and Bone Marrow Infiltration as the First Presentation of Metastatic Signet Ring Cell Carcinoma of Gastric Origin: A Rare Case Report. | Journal of Cancer Clinical Trials | Insufficient patient information |
| Ioannidis | 2012 | Fluoropyrimidine-based chemotherapy as induction, maintenance and rechallenge treatment for gastric carcinomatosis presenting with bone marrow infiltration, microangiopathichaemolytic anaemia and disseminated intravascular coagulation: A case report and l | Forum of Clinical Oncology | Duplicate study data |
| Kadikoylu | 2010 | Thrombotic thrombocytopenic purpura as the first manifestation of metastatic adenocarcinoma in a young woman | Transfusion and Apheresis Science | Wrong patient population |
| Kim | 2008 | Clinical outcome of gastric cancer patients with bone marrow metastases | Oncology | Insufficient patient information |
| Kressel | 1981 | Microangiopathic hemolytic anemia, thrombocytopenia, and renal failure in patients treated for adenocarcinoma | Cancer | Insufficient patient information |
| Lara | 2016 | A rare case of concurrent signet-ring carcinoma of breast and microangiopathic hemolytic anemia | Journal of Surgical Case Reports | Duplicate study data |
| Lara | 2016 | A rare case of concurrent signet-ring carcinoma of breast and microangiopathic hemolytic anemia | Journal of Surgical Case Reports | Wrong patient population |
| Lee | 2004 | A case of bone marrow necrosis with thrombotic thrombocytopenic purpura as a manifestation of occult colon cancer | Japanese journal of clinical oncology | Wrong patient population |
| Lee | 2013 | Aggressive microangiopathic hemolytic anemia associated with metastatic signet ring cell adenocarcinoma of the colon: A note of caution prior to surgical intervention | American Journal of Gastroenterology | Duplicate study data |
| Lee | 2013 | Aggressive Microangiopathic Hemolytic Anemia Associated with Metastatic Signet Ring Cell Adenocarcinoma of the Colon: A Note of Caution Prior to Surgical Intervention | American Journal of Gastroenterology | Insufficient patient information |
| Lee | 2019 | Hemolytic Anemia of Malignancy: A Case Study Involving Signet Ring Cell Metastatic Breast Cancer with Severe Microangiopathic Hemolytic Anemia | Case Reports in Oncology | Wrong patient population |
| Longo | 2006 | Multiorganic dissemination of a colorectal signet ring cell carcinoma with fulminant clinical course | International journal of gastrointestinal cancer | Wrong patient population |
| Mahdi | 2014 | Leucoerythroblastosis and thrombocytopenia as clues to metastatic malignancy | Case Reports | Wrong patient population |
| Mathew | 2008 | Autoimmune thrombocytopenia associated with carcinoma | Journal of Surgery | No MAHA described |
| Mauron | 2000 | Anemia, cutaneous bleedings and loss of weight | Praxis | Insufficient patient information |
| Mendoza | 2012 | Microangiopathic hemolytic anemia complicating disseminated signet ring cell carcinoma in a twenty-year old male: A case report | Phillippine Journal of Internal Medicine | Insufficient patient information |
| Misawa | 2008 | Primary colonic signet ring cell carcinoma presenting carcinocythemia: an autopsy case | Case reports in gastroenterology | Wrong patient population |
| Miyazaki | 2019 | [Carcinoma of the Ascending Colon Showing Rapid Progression of Disseminated Carcinomatosis of the Bone Marrow-Report of a Case] | Gan To Kagaku Ryoho | Insufficient patient information |
| Mizuno | 2009 | Skeletal metastases in gastric cancer: Analysis of skeletal-related events and plasma endothelin-1 | European Journal of Cancer, Supplement | Insufficient patient information |
| Nabeshima | 2003 | Poorly differentiated adenocarcinoma with signet-ring cells of the Vater's ampulla, without jaundice but with disseminated carcinomatosis | Fukuoka Igaku Zasshi - Fukuoka Acta Medica | Wrong patient population |
| Nehme | 2019 | An Unusual Cause of Chest Pain | Gastroenterology | Wrong patient population |
| Ng | 2006 | Malignancy-associated venous thrombosis with concurrent warfarin-induced skin necrosis, venous limb gangrene and thrombotic microangiopathy | Thrombosis and Haemostasis | Wrong patient population |
| Niscola | 2010 | Fulminant thrombotic microangiopathy as a clinical presentation of an occult signet-ring cell carcinoma of the lung and misdiagnosed as idiopathic thrombotic thrombocytopenic purpura | Saudi Medical Journal | Wrong patient population |
| Parekh | 2015 | Bone marrow necrosis discovered in a patient with suspected thrombotic thrombocytopenic purpura | American journal of hematology | Wrong patient population |
| Pendse | 2014 | Hemolytic anemia and metastatic carcinoma: Case report and literature review | Laboratory Medicine | Wrong patient population |
| Ponzo | 2020 | Microangiopathic haemolytic anaemia caused by a signet-ring cell carcinoma of the intrahepatic bile duct |  | Duplicate study data |
| Samie | 2004 | Severe Microangiopathic Hemolytic Anemia as First Manifestation of a CUP Syndrome. Rapid Hematologic Remission under Polychemotherapy. [German] | Medizinische Klinik | Wrong patient population |
| Sato | 1999 | Two cases of myelocarcinoma due to gastrointestinal cancer complicated by microangiopathic hemolytic anemia | Journal of Fukuroi Municipal Hospital | Wrong patient population |
| Shin | 2011 | Microangiopathic hemolytic anemia as the first manifestation of metastatic signet ring cell carcinoma of unknown origin: a case report and review of literature | Korean Journal of Laboratory Medicine | Wrong patient population |
| Wakata | 2006 | Autoimmune thrombocytopenic purpura, autoimmune hemolytic anemia and gastric cancer appeared in a patient with myasthenia gravis | Intern Medicine | Wrong patient population |
| Whittington | 2019 | Signet Ring Cell Carcinoma with Lymphangitic Carcinomatosis in Pregnancy: A Case Report of an Unexpected Maternal Death and Review of the Literature | The American journal of case reports | Wrong patient population |
| Younes | 2017 | Microangiopathic hemolytic anemia caused by a signet-ring cell carcinoma of the intrahepatic bile duct | Minerva Gastroenterologica e Dietologica | Wrong patient population |

**Supplemental Table 6. Tool for assessment of the methodological quality of case reports and case series (adapted from Murad *et al*.)**

| **Domains of Quality Assessment** | **Leading Explanatory Questions** |
| --- | --- |
| **Selection** | Does the patient(s) represent(s) the entire experience of the researchers or is the selection modality unclear to the extent that other patients with similar presentation may have been missed? |
| **Ascertainment** | Was the outcome adequately ascertained? |
| **Causality** | Were other plausible causes that may account for the observation ruled out beyond a reasonable doubt? |
| **Reporting** | Is the case(s) relayed with adequate details to allow other investigators to replicate the research or to permit practitioners to make inferences related to their practice? |

**Supplemental Table 7. Countries of origins for each publication**

| **Country** | **Number of Studies** | **Number of Reported Cases** | **List of References** |
| --- | --- | --- | --- |
| Austria | 1 | 1 | Dempke 2000 |
| Germany | 3 | 10 | Arkenau 2005, Berger 2020, Ferrand 2012 |
| Greece | 1 | 1 | Ioannidis 2009 |
| India | 1 | 3 | Ali 2007 |
| Israel | 1 | 2 | Kaidar-Person 2011 |
| Italy | 1 | 1 | Rossio 2020 |
| Japan | 6 | 14 | Kudo 2015, Matsui 2016, Morimatsu 1985, Oseki 2011, Terasawa 1993, Tsuchiya 1989 |
| Lebanon | 1 | 1 | Otrock 2007 |
| Pakistan | 1 | 1 | Yazdi 2009 |
| Portugal | 2 | 2 | Costa 2018, Pinheiro 2014 |
| Spain | 1 | 1 | Gallegos Sancho 2005 |
| Turkey | 5 | 7 | Ekinci 2020, Gulle 2016, Malkan 2020, Ozkalemkas 2005, Yuce 2016 |
| United States of America | 2 | 2 | Happe 2016, Takayasu 2017 |

**Supplemental Table 8. Assessment of methodological quality of included studies**

| **First author/year (reference)** | **No. Patients** | **Selection**  **(Q1)** | **Ascertainment**  **(Q2)** | **Causality**  **(Q3)** | **Reporting**  **(Q4)** |
| --- | --- | --- | --- | --- | --- |
| Ali 2007 (1) | 3 | 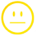 | 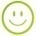 | 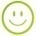 | 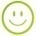 |
| Arkenau 2005 (2) | 1 | 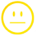 | 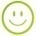 | 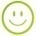 | 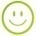 |
| Berger 2020 (3) | 8 | 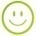 | 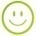 | 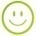 | 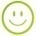 |
| Costa 2018 (4) | 1 | 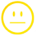 | 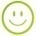 | 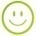 | 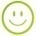 |
| Dempke 2000 (5) | 1 | 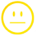 | 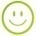 | 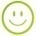 | 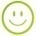 |
| Ekinci 2020 (6) | 1 | 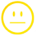 | 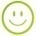 | 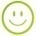 | 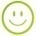 |
| Ferrand 2012 (7) | 1 | 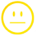 | 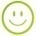 | 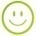 | 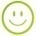 |
| Gallegos Sancho 2005 (8) | 1 | 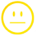 | 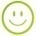 | 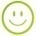 | 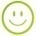 |
| Gulle 2016 (9) | 1 | 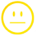 | 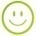 | 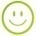 | 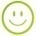 |
| Happe 2016 (10) | 1 | 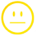 | 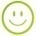 | 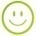 | 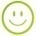 |
| Ioannidis 2009 (11) | 1 | 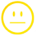 | 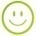 | 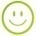 | 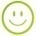 |
| Kaidar-Person 2011 (12) | 2 | 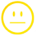 | 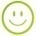 | 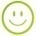 | 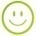 |
| Kudo 2006 (13) | 1 | 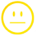 | 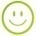 | 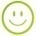 | 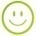 |
| Malkan 2020 (14) | 1 | 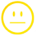 | 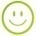 | 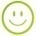 | 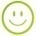 |
| Matsui 2016 (15) | 1 | 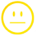 | 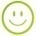 | 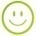 | 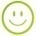 |
| Morimatsu 1985 (16) | 9 | 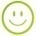 | 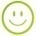 | 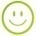 | 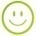 |
| Oseki 2011 (17) | 1 | 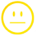 | 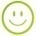 | 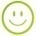 | 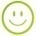 |

| Otrock 2007 (18) | 1 | 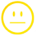 | 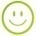 | 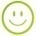 | 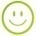 |
| --- | --- | --- | --- | --- | --- |
| Ozkalemkas 2005 (19) | 2 | 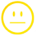 | 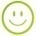 | 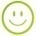 | 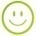 |
| Pinheiro 2014 (20) | 1 | 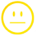 | 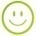 | 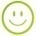 | 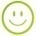 |
| Rossio 2020 (21) | 1 | 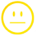 | 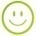 | 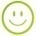 | 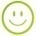 |
| Takayasu 2017 (22) | 1 | 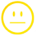 | 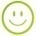 | 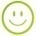 | 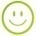 |
| Terasawa 1993 (23) | 1 | 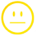 | 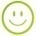 | 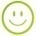 | 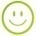 |
| Tsuchiya 1999 (24) | 1 | 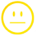 | 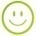 | 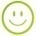 | 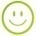 |
| Yazdi 2009 (25) | 1 | 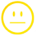 | 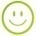 | 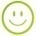 | 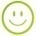 |
| Yuce 2016 (26) | 2 |  |  |  |  |
| **Total: 26 studies** |  | **GMQ 2**  **UMQ 24**  **LMQ 0** | **GMQ 26**  **UMQ 0**  **LMQ 0** | **GMQ 26**  **UMQ 0**  **LMQ 0** | **GMQ 26**  **UMQ 0**  **LMQ 0** |

Good methodological quality – Unclear methodological quality – Low methodological quality.

GMQ: good methodological quality – UMQ: unclear methodological quality – LMQ: low methodological quality.

**Supplemental Table 9. Baseline laboratory values, treatment, and outcome data for 47 cases of signet ring cell cancer of gastric origin and microangiopathic hemolytic anemia**

| **First author** | **Country** | **Age (years)** | **Sex** | **Race** | **Hemoglobin (g/dL)** | **LDH (IU/L)** | **Treatment** | **Follow-up time** | **Final outcome** |
| --- | --- | --- | --- | --- | --- | --- | --- | --- | --- |
| Ali | India | 52 | Female | Asian | 7.2 | NR | NR | 1 week | Death |
| Ali | India | 28 | Male | Asian | 7.5 | 7019 | No treatment | 1 week | Death |
| Ali | India | 21 | Male | Asian | 6.1 | NR | No treatment | NR | Death |
| Arkenau | Germany | 62 | Male | White | 10.7 | 1222 | Chemotherapy | 12 weeks | Death |
| Berger | Germany | 47 | Female | White | 5.9 | NR | Chemotherapy | 10.3 weeks | Death |
| Berger | Germany | 68 | Male | White | 7.7 | NR | No treatment | 0.1 weeks | Death |
| Berger | Germany | 53 | Male | White | 8 | NR | Chemotherapy | 27.1 weeks | Death |
| Berger | Germany | 36 | Female | White | 5.4 | NR | Chemotherapy | 32.1 weeks | Death |
| Berger | Germany | 61 | Female | White | 7.8 | NR | Chemotherapy | 1 week | Death |
| Berger | Germany | 54 | Male | White | 8.4 | NR | Chemotherapy | 28 weeks | Death |
| Berger | Germany | 28 | Female | White | 6.9 | NR | Chemotherapy | 0.3 week | Death |
| Berger | Germany | 76 | Male | White | 8.2 | NR | No treatment | 1.9 weeks | Death |
| Costa | Portugal | 42 | Male | White | 10.2 | 724 | No treatment | 64.5 weeks | Death |
| Dempke | Austria | 48 | Female | White | 9.8 | 467 | Chemotherapy | 8 weeks | Death |
| Ekinci | Turkey | 42 | Male | White | 8.2 | 2826 | NR | NR | Alive |
| Ferrand | Germany | 62 | Female | White | 7.2 | NR | Chemotherapy | 76 weeks | Death |
| Gallegos Sancho | Spain | 34 | Female | White | 8.4 | 1561 | No treatment | 0.1 week | Death |
| Gulle | Turkey | 38 | Male | White | NR | NR | Chemotherapy | NR | Alive |
| Happe | USA | 31 | Female | White | 5.8 | NR | No treatment | 7 weeks | Death |
| Ioannidis | Greece | 71 | Male | White | 6.5 | NR | Chemotherapy | 12 weeks | Alive |
| Kaidar-Person | Israel | 45 | Male | White | 5.9 | NR | Chemotherapy and radiation | 9 weeks | Death |
| Kaidar-Person | Israel | 32 | Female | White | NR | NR | Chemotherapy | 8 weeks | Death |
| Kudo | Japan | 63 | Male | Asian | 10.8 | 898 | Chemotherapy and radiation | 40 weeks | Death |
| Malkan | Turkey | 35 | Male | White | 3.5 | 2257 | No treatment | 4 weeks | Death |
| Matsui | Japan | 68 | Male | Asian | 14.8 | 180 | No treatment | 4 weeks | Death |
| Morimatsu | Japan | 71 | Male | Asian | NR | 160 | No treatment | 8 weeks | Death |
| Morimatsu | Japan | 39 | Male | Asian | NR | 607 | No treatment | 8 weeks | Death |
| Morimatsu | Japan | 35 | Male | Asian | NR | 474 | No treatment | 32 weeks | Death |
| Morimatsu | Japan | 34 | Female | Asian | NR | 650 | Chemotherapy | 10.4 weeks | Death |
| Morimatsu | Japan | 48 | Male | Asian | NR | 3100 | No treatment | 4 weeks | Death |
| Morimatsu | Japan | 49 | Female | Asian | NR | 3700 | No treatment | 12 weeks | Death |
| Morimatsu | Japan | 53 | Female | Asian | NR | 730 | No treatment | 20 weeks | Death |
| Morimatsu | Japan | 46 | Female | Asian | NR | 757 | Chemotherapy | 12 weeks | Death |
| Morimatsu | Japan | 43 | Male | Asian | NR | 758 | No treatment | 24 weeks | Death |
| Oseki | Japan | 75 | Male | Asian | 9.1 | 2063 | No treatment | 4 weeks | Death |
| Otrock | Lebanon | 51 | Female | White | 6.7 | 3350 | Chemotherapy | 12 weeks | Death |
| Ozkalemkas | Turkey | 83 | Female | White | 7.8 | 904 | Chemotherapy | 4 weeks | Death |
| Ozkalemkas | Turkey | 45 | Male | White | 8.4 | 1290 | Radiation | 7.3 weeks | Death |
| Pinheiro | Portugal | 50 | Male | White | NR | NR | NR | 6 weeks | Death |
| Rossio | Italy | 41 | Male | White | 5.4 | NR | No treatment | 1 week | Death |
| Takayasu | USA | 64 | Male | NR | 3.9 | 809 | Chemotherapy and radiation | 2 weeks | Death |
| Terasawa | Japan | 27 | Female | Asian | 7.8 | 746 | NR | 1 week | Death |
| Tsuchiya | Japan | 56 | Male | Asian | 5.7 | 500 | No treatment | 1.7 weeks | Death |
| Yazdi | Pakistan | 19 | Male | Asian | 11.8 | 774 | Chemotherapy | 24 weeks | Death |
| Yuce | Turkey | 34 | Male | White | 9.7 | 774 | NR | NR | NR |
| Yuce | Turkey | 49 | Male | White | 6.4 | 1904 | NR | NR | NR |
| Current report | USA | 36 | Female | Asian | 6.8 | 720 | Chemotherapy and radiation | 11.1 weeks | Death |

NR, not reported

**Supplemental Table 10. Summary of diagnostic workup obtained for pooled patient cohort.**

|  | **Pooled cohort** | |
| --- | --- | --- |
|  | **Number of patients** | **Results** |
| Peripheral blood smear, n (%) | 47 | 33 (70%) |
| Endoscopic diagnosis, n (%) | 47 | 39 (83%) |
| Image Workup, n (%)  CT Scan  MRI Scan  PET Scan  Chest X-Ray  Ultrasound | 47 | 20 (42.5%)  6 (10%)  4 (8.5%)  3 (6.4%)  3 (6.4%) |
| Bone marrow biopsy, n (%) | 47 | 32 (68%) |

**Supplemental Table 11. Summary of upper endoscopic findings**

|  | **Pooled Cohort** | |
| --- | --- | --- |
|  | **Number of patients** | **Results** |
| Endoscopic diagnosis, n (%) | 47 | 39 (83%) |
| Location of gastric lesion, n (%)  Body  Pylorus  Incisura  Cardia | 24 | 13 (54.2%)  5 (20.8%)  3 (12.5%)  3 (12.5%) |
| Endoscopic finding, n (%)  Ulceration  Diffuse Infiltration  Gastric mass | 26 | 15 (57.7%)  8 (30.8%)  3 (11.5%) |

**Supplemental Table 12. Summary of reported details of chemotherapy regimen**

| **First author** | **Country** | **Age (years)** | **Sex** | **Chemotherapy Regimen Details** |
| --- | --- | --- | --- | --- |
| Arkenau | Germany | 62 | Male | ELF Schedule (etoposide 120mg/m^2^, leucovorin 300mg/m^2^, 5-FU 500mg/m^2^ days 1-3, q21), changed chemotherapy regimen after 12 cycles to 5-FU/leucovorin plus oxaliplatin administered bi-weekly |
| Berger | Germany | 47 | Female | 1^st^ chemotherapy line with FLO (5-FU, leucovorin, oxaliplatin); 2^nd^ chemotherapy line with FOLFIRI (5-FU, leucovorin, irinotecan) |
| Berger | Germany | 53 | Male | 1^st^ chemotherapy line with FLO (5-FU, leucovorin, oxaliplatin); 2^nd^ chemotherapy line with Paclitaxel |
| Berger | Germany | 36 | Female | 1^st^ chemotherapy line with FOLFIRI (Leucovorin, 5-FU, Irinotecan)-Ramucirumab |
| Berger | Germany | 61 | Female | 1^st^ chemotherapy line with FLOT (5-FU, Leucovorin, Oxaliplatin, Docetaxel) |
| Berger | Germany | 54 | Male | 1^st^ chemotherapy line with FLOT; 2^nd^ chemotherapy line with FOLFIRI (Leucovorin, 5-FU, Irinotecan)-Ramucirumab |
| Berger | Germany | 28 | Female | 1^st^ line chemotherapy with FLOT (5-FU, Leucovorin, Oxaliplatin, Docetaxel) |
| Dempke | Germany | 48 | Female | Cisplatin 50mg/m^2^ (day 1) and etoposide 100mg/m^2^ (days 1 and 2) started emergently; once there was histological confirmation of diagnosis, chemotherapy switched to 5-FU 2600mg/m^2^ and leucovorin 500mg/m^2^ (days 1 and 8) and then extended to include oxaliplatin 60mg/m^2^ (day 1) |
| Ferrand | Germany | 62 | Female | Modified FOLFOX-6 regimen (oxaliplatin 85mg/m^2^ plus levoleucovorin 175mg/m^2^ over 2h; 5-fluorouracil (5-FU) 400mg/m^2^ as a bolus followed by 2400mg/m^2^ by continuous infusion over 46h repeated q2 weeks for 4 courses; 2^nd^ palliative chemotherapy line with docetaxel; 3^rd^ chemotherapy line with FOLFIRI (Leucovorin, 5-FU, Irinotecan) |
| Gulle | Turkey | 38 | Male | Combination chemotherapy with 5-FU and cisplatin |
| Ioannidis | Greece | 71 | Male | Weekly 24h infusion of high dose 5-FU (2600mg/m^2^) and leucovorin (300mg/m^2^) |
| Kaidar-Person | Israel | 45 | Male | Cisplatin 60mg/m^2^ given IV on day 1 and 5-FU 600mg/m^2^/day given by continuous infusion on days 1-4; cycle was repeated 3 weeks later |
| Kaidar-Person | Israel | 32 | Female | Combination of IV cisplatin (20 mg/m^2^, days 1-5) and a reduced dose of etoposide (60mg/m^2^, days 1-5); 2^nd^ line of chemotherapy line reduced dose of 5-FU (425mg/m^2^, days 1-5) and leucovorin (2mg/m^2^, days 1-5) with weekly dose of cetuximab (average dose 500mg) |
| Kudo | Japan | 63 | Male | S-1 followed by paclitaxel |
| Otrock | Lebanon | 51 | Female | Chemotherapy with cisplatin and 5-FU (cisplatin dose of 50mg/m^2^ biweekly and 5-FU at 150mg/m^2^ weekly); 2^nd^ chemotherapy line with docetaxel and irinotecan |
| Ozkalemkas | Turkey | 83 | Female | One course 5-FU and leucovorin was given |
| Takayasu | USA | 64 | Male | Modified FLOX (oxaliplatin, 5-FU and leucovorin) |
| Current report | USA | 36 | Female | Dose-reduced FOLFOX (oxaliplatin, 5-FU and leucovorin) for 2 cycles |

5-FU, 5-fluorouracil

**Supplemental Figure 1A. Low magnification of the bone biopsy show extensive replacement of the marrow by carcinoma most of which is necrotic (H&E stain, X100).**

**Supplemental Figure 1B. Higher magnification to show small foci of viable cells that are consistent with a poorly differentiated carcinoma similar to Figure 2A (H&E stain, X200).**

**Supplement Figure 1C. Keratin AE1/AE3 immunostain showing the tumor cells to be strongly positive for cytokeratin (X200).**

**Supplemental Figure 2. Esophagogastroduodenoscopy (EGD) of a non-bleeding ulcer located in gastric body.**
